# Spectacles through Charity for School-Children

**Published:** 1906-06-30

**Authors:** 


### Spectacles Through Charity for School-Children.

The appeal recently made to the public, through the *Times* and other channels, for subscriptions in support of a scheme for supplying spectacles to the children at elementary schools, is perhaps the most recent phase of the so-called "charity" which has for many years been steadily tending towards the demoralisation of the industrial classes. In this particular case, the nation has decided that children cannot be suffered to grow up without education; and medical science has shown that the education of children suffering from certain forms and degrees of ametropia cannot be effectively conducted in the ordinary manner unless they are supplied with spectacles. Science, and, in the absence of science, common sense and experience, have also shown that the education cannot be profitably conducted unless the children are sufficiently clothed and fed. The proper persons to supply necessaries to children are their parents; and it is quite certain that, if we except tramps and the great army of unemployables, the industrial classes generally are well able to meet their responsibilities of this nature, and, as a rule, are willing as well as able. But there are some who demur; and at this point the philanthropist steps in. Instead of punishing the negligent parent, and of affixing some stigma, such as the loss of the franchise, to his default, he proposes to find the food or the spectacles himself. As soon as this tendency has been made sufficiently manifest, it acts upon an enormous number of people who would previously have been quite prepared to discharge their parental duties. They will argue that, if they do not provide the food or the spectacles, somebody else will, and that they themselves will have the price of another pint. They are not so much criminals as weak and self-indulgent human creatures, quite ignorant of the extent and character of the evils which their conduct may entail upon their offspring, and quite ready to do their duty if the laxity of their moral fibre receives a little stiffening from legislation and from public opinion.

The circular informs us that the wholesale opticians who are to be employed will supply spectacles with spherical lenses for tenpence a pair, and with cylindrical lenses at prices starting from half-a-crown. The "Committee" are of opinion that glasses at these prices are within the reach of all but the "very poor," and that the charity might be "almost" self-supporting. They propose to enlist the services of as many hospitals as will fall into the scheme, and they contemplate the possibility of enabling the children to obtain "cheap and excellent spectacles." They expect, however, to require an income of £200 a year; money enough, that is, to provide sixteen hundred pairs of cylindrical or four thousand eight hundred pairs of spherical spectacles; and it is plain that these or their equivalents are intended to be given away to somebody. The question naturally arises, to whom? and under what guarantee? We have no fault to find with an organisation which will assist parents to obtain glasses for their children at a proper price, and to secure that they are of a kind suited to each child's requirements; and, as far as the arrangements with hospitals are concerned, and the nomination, in each district, of a dealer in spectacles who is approved by the County Council, and is prepared to supply prescribed glasses at a definite scale of prices, we have nothing but approval of the scheme. We see no reason why it should cost £200 a year, or why it should require any Committee outside the Council itself. If the Council has no authority, there is at present an Education Bill before Parliament, into which it ought not to be impossible to introduce one rational and non-contentious clause. It is at least certain that, if the parents of children requiring them were compelled to provide glasses, the glasses would be taken better care of than if they are given away. The number to be provided would be considerable; and the prices quoted by the Committee in the circular before us would allow dealers a very fair margin of profit upon a large transaction. Mere glasses are cheap enough, insomuch that excellent spectacles, as far as the glasses alone are concerned, can be bought wholesale at sixpence a pair, or with cylindrical glasses at one and ninepence. The weak point in cheap spectacles is the frame, which is seldom calculated to bear the rough usage it would be likely to receive from children. It is with regard to the frames, generally speaking, that the wares supplied under contract would require close and constant supervision, not only with regard to their actual strength and quality, but also with regard to the proper fitting of that which was supplied to each child.

